# Improving Understanding of and Adherence to Pulmonary Rehabilitation in Patients with COPD: A Qualitative Inquiry of Patient and Health Professional Perspectives

**DOI:** 10.1371/journal.pone.0110835

**Published:** 2014-10-30

**Authors:** Su-Er Guo, Anne Bruce

**Affiliations:** 1 Chronic Diseases and Health Promotion Research Center and Research Center for Industry of Human Ecology, Chang Gung University of Science and Technology, Chiayi, Taiwan; 2 School of Nursing, University of Victoria, Victoria, Canada; University of Dundee, United Kingdom

## Abstract

**Objectives:**

Although patients with Chronic Obstructive Pulmonary Disease (COPD) who adhere to a pulmonary rehabilitation program are better able to manage their illness and experience a better health-related quality of life, pulmonary rehabilitation remains underused. This study aims to describe the experiences of patients who are in a pulmonary rehabilitation program, and explore the perceptions of both patients and health professionals about what improves effective pulmonary rehabilitation.

**Methods:**

A qualitative research design, including focus groups and individual interviews with 25 patients and 7 program health professionals, was used to obtain combined perspectives about the factors underpinning the COPD patient's reasons for participation in a rehabilitation program.

**Results:**

Three themes were derived from the descriptive content analysis: (1) building confidence, (2) a perception of immediate tangible results, and (3) being ready and having access to the program.

**Conclusions and Practical Implications:**

Qualitative findings from this study suggest that a patient's adherence to a COPD rehabilitation program can be improved by quickly building up the participant's confidence, promoting tangible results, and by timely recognizing and responding to the issues of readiness and access. Based on these findings, health care providers could develop strategies to better serve COPD patients who face multiple barriers to access and successfully complete a pulmonary rehabilitation program.

## Introduction

Chronic obstructive pulmonary disease (COPD) is a major cause of disability and death in Canada and throughout the world. It was reported in 2012 to be the third leading cause of death in the world [1]. COPD rates are expected to continue to rise steeply with an ever increasing mortality rate [2], [3]. This chronic disease resulted in an estimated economic cost of USD 2.1 trillion in 2010 and is projected to cost 4.8 trillion in 2030 [4]. It is clear that the efforts to improve the health of those living with COPD need closer attention.

In response to the rising morbidity and mortality associated with this disease, a pulmonary rehabilitation program was developed to optimize the prevention and management of COPD [5]. Pulmonary rehabilitation (PR) is a multidisciplinary approach to optimizing the physical and social functioning as well as the autonomy of the patients. PR offers the best chance to manage the symptoms and reduce health resource use [6], [7]. Recent research indicates that PR programs increase health related quality of life in patients with COPD [6], [7], [8], [9]. Nevertheless, available PR programs remain underused by COPD patients [10], [11].

Some studies investigated the adherence by COPD patients to PR programs. Brooks et al. [11] surveyed the Canadian PR programs and compared their findings with similar works conducted in the late 1990s [12]. Their research was mostly descriptive and examined the general characteristics of the various programs and their participants, including location, size and duration, content, staffing, and the type of referral [11]. More complex issues related to patient enrolment and attendance however were not addressed. Most of the studies examining PR, tend to focus on the rate and the predictor of adherence [13], [14]. Some recent qualitative studies have shed light on the PR attendance of patients with COPD [15], [16], [17]. In one study, researchers used qualitative interviews with 19 COPD patients who had declined to participate and 18 COPD patients who had not completed a PR program to understand their experiences of the PR uptake and completion [15]. Findings reveal a lack of perceived benefit from the PR program and inconvenient transport were significant concerns for patients [15]. Other researchers identified factors from COPD patients concerning PR attendance and completion, such as group support, self-confidence, the influence of the referring physician [16], fear of being breathless and exacerbation of existing medical problems [17]. Of note, while only a few studies explore patients' perspectives, no literature was found that explored perceptions of PR attendance from the health professionals' perspective working in PR programs. Given the lack of qualitative description regarding the experience of COPD patients following a PR program and the perspective of professional clinical staff, this study aimed to explore patients' and health professionals' perceptions of the attendance and completion of PR, within the context of outpatient PR programs. The overall goal was to better understand the experiences and thoughts attributed to PR attendance and to identify barriers and strategies to establish effective pulmonary rehabilitation.

## Materials and Methods

A qualitative descriptive research design was used to obtain multiple perspectives about factors underpinning the reasons COPD patient participate in a rehabilitation program. Data were generated from four sources: (1) qualitative focus groups with COPD participants, (2) focus groups with program facilitators, (3) individual interviews with COPD participants.

### Ethics Statement

This study was approved by the research ethics boards of both the University and the Health Authority where this study was located. As per standard PR program protocol, PR staff members schedule an initial assessment session with each COPD patient referred to the program by a physician. At the end of the assessment session the PR staff member who conducted the session asked the patient if they would like to learn about a PR-related research study. If the patient was interested in learning more, the PR staff member referred them to research assistant. All participants were briefed by the researcher or research assistant regarding the research purpose, methods, and involvement required. Prior to participant enrollment, written consent was obtained. Health care professionals were purposively sampled through invitations to participate from a third party or flyers in accordance with procedures approved by the research ethics boards.

### Study Participants

The study included COPD participants (N = 25) from three different PR programs offered by two different hospitals and one community center in a large Canadian city. Here the eligibility criteria stipulated adults 40 years of age or older, diagnosed with COPD, referred by a health professional, having completed at least 75% of the PR program and being able to read and speak English. Patients with other organ dysfunction, skeletal muscular disorders, cancer, or who were unable to cooperate were excluded from the study. The other sample group consisted of program health professionals (N = 7) currently staffing PR programs at the three sites in the catchment area that included physiotherapists, respiratory therapists and registered nurses.

### Description of the Three PR Programs

Participants were recruited from three PR programs in the catchment area. All three programs were outpatient, multidisciplinary programs, each offering 24 or more consecutive sessions. Each session included both education and exercise training. The education sessions addressed the anatomy of the respiratory tract and the physiology of COPD, the effects of smoking, and also provided self-care skills. A multidisciplinary team consisting of a chest physician, nurse, physical therapist, and a dietician provided care and advice during each session. After completing the PR program no other structured and/or supervised home exercise programs were offered, but patients could choose to participate in a maintenance exercise program.

### Data Collection

Four to six weeks after the end of the program, an audiotaped focus group interview was conducted with the COPD PR participants from each of the three PR programs on site. To ensure richness of data, three participants were selected (one from each group) to be invited to take part in an individual interview. The selection criteria included being expressive and willing to share their experiences of living with COPD and attending a PR program. The interviews were conducted by telephone and audio-taped, and included an elaboration of the topics identified in the earlier focus group. The other focus group consisted of 7 professional PR staff, including three respiratory therapists, two physiotherapists and two registered nurses from among the three PR programs where focus groups were recorded.

### Focus Groups

A total of 25 COPD patients across three sites participated in four focus groups and 7 health professionals participated in one group. All focus groups followed the procedure as outlined by Kreuger and Casey (2000) [18]. Semi-structured questions were used to seek information about what social, psychological and environmental factors affected patient participation in a PR program. The questions included: How did you come to being referred to a PR program? What helped you the most with completing the PR program? Why do you think some people do not attend a PR program? Generally speaking, which factors do you feel can help people to finish the program? The health professionals were asked similar questions such as: In your experience, what are the underlying reasons for COPD patients not to complete a PR program? What do you believe is the reason behind the decision of a patient not to enroll in the program? In your opinion, what works well to keep participants interested in completing a pulmonary rehabilitation program? (Table 1)

**Table 1:** Semi-structured questions covered in the participant interview.

COPD Patients	PR Program health professionals
How did you come to being referred to a PR program?	In your experience, what are the underlying reasons for COPD patients not to complete a PR program?
What helped you the most with completing the PR program?	What do you believe is the reason behind the decision of a patient not to enroll in the program?
Why do you think some people do not attend a PR program?	In your opinion, what works well to keep participants interested in completing a pulmonary rehabilitation program?
Generally speaking, which factors do you feel can help people to finish the program?	

Note. COPD: Chronic Obstructive Pulmonary Disease; PR: Pulmonary Rehabilitation.

### Data Analysis

Transcribed data were coded using a thematic analysis approach [19]. Specific analytic steps included: (1) Focus group data were transcribed verbatim and checked for accuracy against the taped recordings. (2) Transcripts were read several times by both investigators to identify recurring, converging and opposing themes and patterns. Patient transcripts were read separately followed by close reading of health professional perspectives. Finding no contradictory views, transcripts were coded together for themes of patient experiences and what improves effective pulmonary rehabilitation. Key concepts and illustrative examples from the data were identified. (3) Preliminary concepts and themes were compared and collapsed into overarching themes and sub-themes.

### Trustworthiness

Guba and Lincoln (2005) proposed that in qualitative inquiry, researchers pursue rigor by establishing the trustworthiness of the interpretations [20]. An integral aspect of trustworthiness is maintaining a detailed audit trail. We recorded our reflective memos and data analysis decisions throughout the analysis. Themes were identified independently and then followed up by a consensus, reached through discussions between researchers to establish the central themes. Confirmability was enhanced by means of using analysis and process notes to avoid a potential bias [21].

## Results

Twenty-five COPD patients (moderate to severe COPD without chronic respiratory failure) were enrolled. Diagnosis of COPD was made according to the International GOLD guidelines [22]. At the time of recruiting, patients were all in stable condition. Table 2 shows the characteristics of the participants.

**Table 2:** Patient Characteristics.

Characteristics	n	%
Gender		
Female	12	48.0%
Male	13	52.0%
Race		
Caucasian	23	92.0%
Asian Canadian	2	8.0%
Retired	24	96.0%
Smoking status		
Non-smoker	16	64.0%
Experimenter	1	4.0%
Current smoker	8	32.0%
Severity of COPD		
Moderate (80<FEV_1_ predict ≦50)	7	28.0%
Severe (50<FEV_1_ predict ≦30)	12	48.0%
Very severe(FEV_1_ predict<30)	6	24.0%

	Mean±SD	Range
Age (years, Mean±SD)	68.8±7.7	53–84
Education (years, Mean ±SD)	12.8±2.1	9–16
BMI (Mean ±SD)	27.7±5.8	17.9–40.6
FEV_1_ predict (%, Mean ±SD)	47.6±15.4	24–77

Note. SD: Standard Deviation.

(N = 25).

Three themes were generated using the process outlined above. Table 3 depicts the themes and their constituent components or sub-themes. Key themes include, *building confidence*, *perceiving immediate tangible results, readiness and access*.

**Table 3:** Focus group themes and subthemes.

Themes	Subthemes
Building Confidence	Learning to live with COPD and limitations
	Regaining independence and agency
	Learning practical techniques
	Experiencing positive group dynamics- fun
Perceiving immediate tangible results	Gaining relevant knowledge
	Individual attention in first few classes
Readiness and access	Perceiving need and benefits
	Working with fear
	Developing diverse entry points
	Minimizing access barriers

### Building Confidence

Being diagnosed with COPD was frightening for many participants. It made them feel vulnerable, concerned and undermined their confidence. Some participants had multiple co-morbidities complicating or delaying the diagnosis, while others knew that something was wrong but deferred seeking professional help. Their needs were described by a PR staff member as “*huge, with everything from filling out financial aid and disability forms, to dealing with loneliness, depression and anxiety.*” As a result, some participants became progressively more isolated and depressed and less mobile. As one patient mentioned, “*when I was finally diagnosed with COPD I didn't believe it, but since I had trouble breathing there had to be something wrong…I gave up golf and I stopped going fishing. In fact, I became a hermit and pretty much just sat in my den most of the day.* ” Another patient stated “*I've lost 50% of my lung capacity to this. I've got emphysema, asthma and bronchitis. My heart rate is way too high and my blood pressure is sky high, so I am just living from day to day.*” PR staff noted that people who really need pulmonary rehabilitation are often physically limited and lack confidence in their ability to improve their quality of life.

Motivations for joining a PR program were mixed and affected the decision of the participant to register with a program and whether or not to complete it. Several patients were initially fearful or demoralized and were of the opinion that their disease was too far advanced to be able to benefit from rehabilitation–as one patient shared, “*I wasn't just short of breath, I was gasping for breath.*” These patients expressed misgivings about the program and were unaware of the potential for a better quality of life with COPD. However PR staff also identified people who were highly motivated and had taken the initiative, including asking a physician for a referral. While some patients were less confident in their ability to improve their quality of life, some were more optimistic as shared by one patient who wanted to “*learn everything that can help me*”.

Across this continuum participants reported that the knowledge and skills offered by the PR program helped them to realize that COPD was not a death sentence, but something that they could learn to manage effectively. An important outcome of the program was learning how to live with the limitations imposed by COPD. Participants spoke of regaining independence. As one patient mentioned, “*When I go out, I try not to walk too far*. *I know my limits, and it makes life easier.*” Another participant shared, “*Now I'm no longer so nervous. I know when I need to sit down and do my deep breathing exercise. Now that my friends know that there are things I can do to get my breath, they are more comfortable with, and that makes it easier for me*”. There was a greater acceptance of the progressive nature of the disease: “*If I can't change the illness then at least I can learn how to happily live with it*.”

Key factors in committing to a program were the relationship and the group support fostered through the program as described by a patient, “*You are with people who understand, because they are walking the same walk*”. A patient spoke positively of group dynamics and co-learning, “*because he had been in the program before, he was really informative with tidbits like how to live with an air conditioner*.” PR staff paid close attention to relational approaches to teaching and learning and organized their classroom seats in circles, encouraging story-telling approaches, and built time for socializing into the schedule. In addition to providing social support, staff encouraged participants to make a concerted effort to build their confidence from the very beginning of the program by paying close attention in the first few classes, thereby creating pragmatic learning opportunities. As one staff member put it; “*we consistently see that people can change and can make a difference. If we can get them to believe that, then that is helpful*.”

### Perceiving Immediate Tangible Results

The PR programs varied in structure and length. Of the three programs, one used a six-week model with 2 sessions per week followed by an optional six-week maintenance program. The others used a one-month model with 3 sessions per week without an optional follow-up maintenance program. All programs included an assessment interview that as one RN stated was vital for offering “*A little hook to people to see some value in their daily life*”.

*“…if we can give them something in that initial interview that they can see gaining some benefit from, then they're more inclined to come back. Sometimes it just means showing them how to correctly use their medications, or show them how to use pursed lip breathing when bending over to put on their shoes and socks….”*

Despite the difference in structure and intensity, participants across these 3 programs indicated that having positive results immediately was what gave them most of the motivation to continue. In particular, learning techniques for breathing and walking resulted in unexpected experiences of well-being, “*in about ten minutes she taught me how to go upstairs and now I have no problem, none*.” Or as another patient shared,

*I would be tired, crawling up the 10^th^ step of the stairs and so she came stand beside me while one of the instructors sat beside me. She had already told me how to breathe and I couldn't believe that I got up to the top and I wasn't winded at all. It was like I hadn't walked any stairs.*

Another patient stated, *“If I hear of anybody that's got any type of COPD I say, well you should talk to your doctor about getting into that program–Cause it works”.* The tangible change in people's mobility was seen as a simple but dramatic shift, but that it required expert input.

Learning about pathophysiology, understanding the disease process and how the medications worked was also valued. One PR staff member observed, “*People weren't using their puffers because the doctor kind of glazed over why and how to use it. Having someone take the time to go through it step by step was well worth it to them*”. Program participants responded well to measures illustrating “*signs of progress*.” In one clinic, oxygen levels were measured using pulse oximeters before and after activity sessions of walking or bicycling, followed by a five-minute rest break. Participants then assessed how oxygenated their blood was. They found these tracking methods interesting and motivating. In addition to pulse oximeters, others liked having large clocks visible so that they could keep track of their walking or cycling speeds to check on their progress or decline each week. The strategies put in place to highlight or enhance tangible and positive results were identified as strong factors that helped them to complete the program.

### Readiness and Access

Participants spoke of the need to be mentally and physically ready before attempting to successfully complete the program. For some this was their second or third time participating in a PR program because previously they were not able to commit themselves fully due to illness. Illnesses, such as colds, for COPD patients can take weeks to resolve and may undermine their readiness to attempt another program. Furthermore, some patients might not fully commit themselves because of what one staff member referred to as “*a lackadaisical attitude*.” PR staff conceded that patients “*have to want to work at their own health; they have to be responsible for their own health*.” Where a minority of patients was seen as not wanting to put in the required effort of doing regular exercises or “*wanting a magic pill that was just going to fix them*”, fears and concerns were identified as more significant barriers for the majority of the participants. As one woman stated, “*When I get anxious, then I know that I can't breathe properly and can*'*t get enough air. It is almost like you have to get bad enough to be able to realize that ‘oh I really need this’ before you use it*”. Fear of exercise and shortness of breath, discomfort with the notion of going to “*the gym*”, and concerns of contracting germs when attending a hospital-based program were also identified as impeding participants' readiness to participate in or complete a PR program.

One physiotherapist explained that many patients are hesitant to exercise because “*they think it's beyond them*”.

I think that generally speaking, if you said to a group of 70 or 80 year olds….” *OK, let's go and do some exercise, they would be horrified, not to even mention those that would be short of breath from just getting out of bed or their easy chair to go to the toilet or making themselves a drink. The thought of exercising I think is just beyond their comprehension.”*

Another factor is that some patients believe that having shortness of breath is detrimental to their lung capacity. As one RT shared, “*they seem to think that [exercise] could be damaging their lungs and they don't want to get worse. They may not necessarily want to get better but they surely don't want to get worse*.”

#### Concerns regarding access

There were also multiple access issues demonstrating that readiness was not only an individual choice but that it also had systemic components. Practical issues such as finances, parking, transportation and psychosocial support shaped how much people were able to participate. For example, one PR program included a second six-week program at an added cost of $40 which was prohibitive for some patients on a fixed income, especially when parking costs were high. As one staff member explained, “*When you have to pay for parking, which I think is six dollars an hour right now at the hospital, and when you come a little early to find a parking spot by the time you get out, it's like three hours and that is twice a week for 12 weeks, that gets to be costly*”. One site used a sliding scale approach for program costs but transport was a key issue. For many, public transport was not a viable option because participants were often unable to walk to a bus stop and then up a hill as was required. PR staff also noted that people did not always know about the availability of transportation services for people with disabilities.

## Discussion

The results of this study revealed that successful adherence to PR programs among these Canadian patients with COPD was associated with building up the confidence of the participants, having them perceive immediate tangible results, and recognizing and responding to patient readiness and providing ready access.

Attending pulmonary rehabilitation is a challenge for most COPD patients since most have shortness of breath and are activity intolerant. Most people will avoid any negative experiences of their symptoms and actively choose inactivity rather than exercise. This is especially the case for COPD patients who do not realize or understand that exercise helps to improve their health. Even when told that exercise will help, they may not believe it. Several studies have suggested that patients adhere to an intervention if they perceive that they will receive benefits from that intervention [15], [17], [23], [24], [25], [26]. In addition, the impact of coexisting medical problems [17], [24] and transport issues on pulmonary rehabilitation are common concerns and have been previously reported [15], [25], [27], [28], [29]. On the contrary, this study revealed that if patients are not ready or do not see immediate tangible results, they might do other things and skip the PR programs. Therefore, in clinical practice, professionals need to give patients some clues, time, space, and opportunity to identify and experience the advantages and benefits of exercise. Findings from this study also suggest that people need to be introduced slowly to the idea of exercise (pulmonary rehabilitation). Most people need to see the advantages and benefits of a PR program before they will consider starting and possibly completing a program. Participants tended to attend a PR program at their own pace, based on their lifestyle habituated and limited by the disease. This finding is similar to previous research showing that most people try to find a way to regain their life and live with COPD and its limitations [17]. It's a process they have to work up to, and not everyone accomplishes that at the same pace.

A key factor that encouraged patients to complete a program was confidence and group support. This finding reflects Bandura's Social Cognitive Theory (1986) which suggests that self-efficacy is an important factor for behavioral change [30]. Self-efficacy, like confidence, refers to a belief in one's capabilities to successfully execute a course of action. Several studies have shown that self-efficacy is a determinant of exercise adherence [31], [32], [33], [34]. Self-efficacy is informed by social and professional support. Aronld, Bruton, and Ellis-Hill (2006) documented how the referring doctor plays a key role [16]. Unlike their finding, the present study found that readiness and group support, rather than the impact of a doctor's advice, were the key factors in completing a PR program.

Findings from this study about adherence to PR programs also align with the Transtheoretical Model (TTM) of change which assesses five stages of readiness including precontemplation, contemplation, preparation, action, and maintenance to change behaviour [35], [36], [37], [38], [39]. For example, two themes of concern for participants in our study were ‘Building Confidence ’ and ‘Seeing tangible results quickly’ which are congruent with stage two (Contemplation, Getting Ready) and stage three (Preparation (Ready) of TTM. Another theme, ‘Readiness and Access’ falls between stage three (Action) and stage four (Maintenance). Based on these congruencies, health care professionals can create environments and experiences to support patients' readiness for change. Such strategies could include helping patients see tangible results quickly and building their confidence in order to facilitate their initiative to change. Therefore, health care professionals are encouraged to articulate clearly what change will look and feel like if a COPD patient was to attend pulmonary rehabilitation. They need to clearly explain why pulmonary rehabilitation is important and highlight the potential benefits so that they provide a vision of a better future to the patient. These approaches are similar to the strategies, reinforcement management and helping relationships as recommended by TTM [40].

### Practical Implications

The PR participants and the staff made several recommendations to boost registration and get patients to complete the program by 1) Making a referral for PR earlier on in the illness trajectory; 2) introducing the PR option and explaining it to hospitalized patients; and 3) linking PR programs with existing community-based programs.

### Making the Referral Earlier

While educational programs have been implemented to inform GP's about the availability of PR programs, there is, as one participant mentioned, “this ongoing occasional issue about a lack of referral and yet we know that they (GP's) see people with COPD.” In addition to making referrals earlier, PR staff also suggested that adequate planning was needed by the program coordinator to ensure proper funding, appropriate screening of the applicants, and to ensure adequate group sizes. Johnston et al. (2013) examined the barriers and the facilitators for being referred to a PR program for COPD patients in Australia [41]. The authors interviewed GP's regarding their perception of the challenges faced by COPD patients in attending a rehabilitation program. The perceptions identified by the GP's reflect the findings from our study– namely, difficulties with transport, lack of perceived benefit, and not being well – as reasons for not registering in an offered PR program. Other findings reported by Johnston et al include perceptions that GP's offered sufficient advice.

### Introducing PR to Hospitalized Patients

A respiratory therapist in this study recommended using the term “lung attack” for notifying medical staff about a COPD patient with an exacerbation that required hospitalization. Then, similar to cardiac patients who are sent to cardiac rehabilitation, these COPD patients should then be considered for pulmonary rehabilitation.

### Linking PR Programs with Existing Community-Based Programs

One of the three programs reviewed in this paper was linked with an existing Better Breathers Club and the YMCA. Anecdotally, PR staff found that community integration encourages patients to become more independent and less reliant on health professionals. Integration with community-based programs was seen as a means to sustain the life-style changes for patients who did not exercise before and needed continued group support beyond the four or six week PR program. Building up a patient's confidence allowed them to continue with their exercise program on their own, once the program was complete. It was seen as “preventative care” that would provide financial savings to the health care system in the long run. It was also suggested that patients should have the flexibility to be able to take the program over again if they were unable to continue a regular exercise practice on their own. Finally, staff recommended that an exercise maintenance program should be provided once or twice a year so that the long-term benefits to the patients could be tracked.

### Limitations

As with all qualitative research, we are unable to generalize findings from this research since the sampling strategy is purposive rather than random. However, the findings can be used by clinicians to better understand perspectives of some COPD patients and PR health professionals. Readers may also recognize themes that represent situations in their contexts to inform their practice.

## Conclusions

Pulmonary rehabilitation is an important secondary prevention for individuals with COPD. In addition, patients with COPD who complete a PR program are reported to manage their illness well, and they enjoy a better health related quality of life [7]. However, PR is persistently underutilized, and those that do attend a PR program often do so partially. Qualitative findings from this study suggest that adherence to COPD rehabilitation programs can be enhanced by building participant confidence quickly, fostering tangible results, and recognizing and adjusting to the issues of readiness and access. Based on these findings health care providers can develop strategies (such as “inform patients about any changes or improvements”, “develop Q & A session on space and time”, “establish a support group”) and approaches to better serve COPD patients who face multiple barriers to access and successfully complete a PR program.
